# Micro‐Gel Ensembles for Accelerated Healing of Chronic Wound via pH Regulation

**DOI:** 10.1002/advs.202201254

**Published:** 2022-05-21

**Authors:** Tingting Cui, Jiafei Yu, Cai‐Feng Wang, Su Chen, Qing Li, Kun Guo, Renkun Qing, Gefei Wang, Jianan Ren

**Affiliations:** ^1^ State Key Laboratory of Materials‐Oriented Chemical Engineering College of Chemical Engineering Jiangsu Key Laboratory of Fine Chemicals and Functional Polymer Materials Nanjing Tech University Nanjing 210009 P. R. China; ^2^ Department of General Surgery Jinling Hospital Nanjing Medical University Nanjing 210002 China

**Keywords:** accelerated wound healing, chronic wound, microfluidic assembly, micro‐gel ensembles, wound pH regulation

## Abstract

The pH value in the wound milieu plays a key role in cellular processes and cell cycle processes involved in the process of wound healing. Here, a microfluidic assembly technique is employed to fabricate micro‐gel ensembles that can precisely tune the pH value of wound surface and accelerate wound healing. The micro‐gel ensembles consist of poly (hydroxypropyl acrylate‐*co*‐acrylic acid)‐magnesium ions (poly‐(HPA‐*co*‐AA)‐Mg^2+^) gel and carboxymethyl chitosan (CMCS) gel, which can release and absorb hydrogen ion (H^+^) separately at different stages of healing in response to the evolution of wound microenvironment. By regulating the wound pH to affect the proliferation and migration of cell on the wound and the activity of various biological factors in the wound, the physiological processes are greatly facilitated which results in much accelerated healing of chronic wound. This work presents an effective strategy in designing wound healing materials with vast potentials for chronic wound management.

## Graphical abstract

Micro‐gel ensembles are developed to allow continuous and intelligent regulation on wound microenvironment pH over the four healing phases of hemostasis, inflammation, hyperplasia, and remodeling, which can effectively promote the formation of new blood vessels, the proliferation and migration of fibroblasts and adipocytes, and the polarization of macrophages, and ultimately accelerate wound healing.

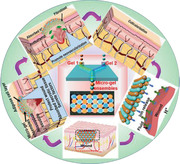

## 1 Introduction

Skin wound healing is one of the most complicated physiological processes in the human body.^[^ ^1^ ^]^ Normaly, such healing process includes four overlapped phases, namely, hemostasis, inflammation, hyperplasia, and remodeling. The continuous completion of the four phases is a necessary condition for effective wound healing and restoration of wound microenvironment balance.^[^ ^2^, ^3^ ^]^ To restore the integrity of the skin upon the four phases, the spatial‐temporal synchronization of multiple cell types is involved, including inflammatory cells, fibroblasts, keratinocytes, macrophages, adipocyte, and so on.^[^ ^4^, ^5^ ^]^ Besides, metabolism and proliferation of wound cells during the healing process are greatly affected by wound microenvironments (e.g., physical, chemical, and biological factors),^[^ ^6^, ^7^ ^]^ such as temperature,^[^ ^8^ ^]^ pH of the wound site,^[^ ^9^, ^10^ ^]^ oxygen level,^[^ ^11^ ^]^ or glucose level.^[^ ^12^ ^]^ Accordingly, an increasing number of wound treatment strategies have been designed, such as phototherapy,^[^ ^13^ ^]^ wound dressing treatment,^[^ ^14^ ^]^ tissue transplantation,^[^ ^15^ ^]^ extracellular matrix (ECM) and cell‐based therapies,^[^ ^16^ ^]^ and genetic engineering.^[^ ^17^ ^]^ However, the vast majority of these approaches mainly focus on a specific stage of the four phases to realize wound healing.^[^ ^18^, ^19^ ^]^ If we can ensure the continuous and effective regulation over the whole four phases for wound healing, accelerated healing and the restoration of some tissue functionality are possible.

As one of the essential parameters of wound microenvironments, intracellular and extracellular pH potently influences cellular processes (enzymatic activity, macromolecular synthesis, transport of metabolites) and cell cycle processes (inflammation, collagen formation, and angiogenesis) over the four phases.^[^ ^12^, ^20^ ^]^ Intact skin is naturally acidic with pH value ranging from 4 to 6. Upon injury, the pH value of the wound surface increases owing to the leakage from the microvessels, and approximates a physiologic pH (7.4) accommodating to bacterial infections.^[^ ^21^ ^]^ Consequently, the pH value becomes more alkaline, commonly between pH value 7.5–8.9, which causes inflammation, and leads to prolonged wound healing.^[^ ^12^ ^]^ When rebuilding the barrier function in chronic wounds, restoring the acidic environment of the wound can reduce the microbial load on the skin surface and reduce the susceptibility of bacterial colonization.^[^ ^22^ ^]^ And the recovery of the acidic environment can also help adipose tissue to restore metabolism.^[^ ^23^ ^]^ Nevertheless, the proliferation and migration of keratinocytes and fibroblasts prefer an alkaline milieu with pH value around 8.3.^[^ ^24^, ^25^ ^]^ Therefore, appropriate regulation on the wound pH during the four phases may enable accelerated wound healing,^[^ ^26^ ^]^ for instance, building an acidic environment at initial stages (hemostasis and inflammation) to inhibit bacterial infection and promote vascular regeneration, and an alkaline environment there after (hyperplasia and remodeling) to promote cell proliferation and encourage skin remodeling. However, continuous wound pH regulation over the four phases remains challenging and has yet to be achieved.

Hydrogels have received increasing attention in the past decades for biomedical application.^[^ ^27^, ^28^, ^29^ ^]^ They are also widely employed as wound dressing because their unique 3D network structure can hold large amounts of water and provide an ideal living environment for cell growth.^[^ ^30^, ^31^, ^32^ ^]^ Besides, in light of the importance of pH to wound healing, researchers have developed various pH‐responsive hydrogels to monitor the pH of chronic wounds,^[^ ^12^, ^33^ ^]^ which are helpful for determining the effective treatment strategies. However, few studies have considered from the perspective of hydrogel regulating wound pH to achieve wound healing.

**Figure 1:**
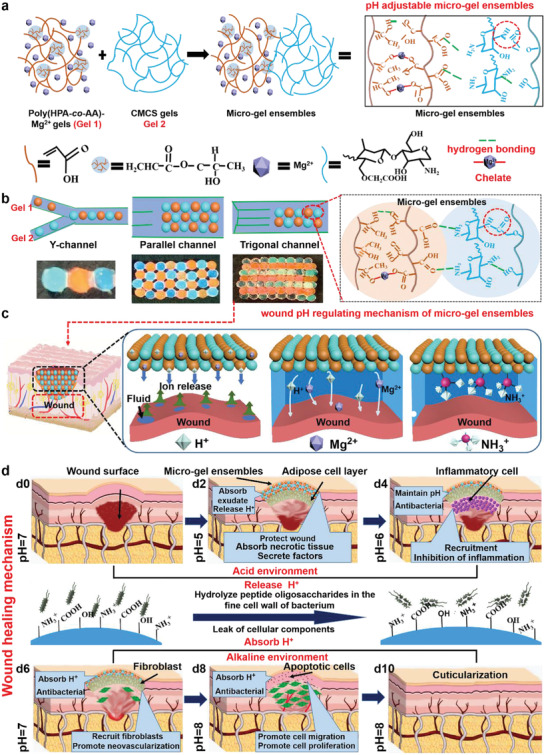
Formation and application of micro‐gel ensembles. a) Schematic synthesis of micro‐gel ensembles via hydrogen bonding of Gel **1** and Gel **2**. b) Microfluidic assembly of Gel **1** and **2** into micro‐gel ensembles with various macrostructures using specific microfluidic chips and channels. c) The pH regulating mechanism of micro‐gel ensembles for skin wound treatments, and d) the corresponding skin healing mechanism. Initially, the ‐COOH group on the surface of Gel **1** releases free H^+^ into the wound microenvironment, which adjust the pH of the wound during the early stage of skin healing. Then, the rich ‐NH_2_ group in Gel **2** absorbs free H^+^ from the microenvironment, converting to NH_3_^+^ and destroying the bacterial membrane structures, thus protecting the wound whilst regulating the pH value of the wound during the late stages of skin healing. Finally, micro‐gel ensembles regulate the wound's microenvironment, facilitating rapid healing process (anti‐infective, adipocyte covering wound, and macrophage polarization).

Herein, we develop micro‐gel ensembles capable of continuously and intelligently regulating wound microenvironment pH over the four healing phases, which could greatly facilitate the healing of chronic skin traumas and the restoration of tissue functionality. Two biocompatible hydrogels with decent mechanical robustness, poly (hydroxypropyl acrylate‐co‐acrylic acid)‐magnesium ion hydrogel (poly (HPA‐*co*‐AA)‐Mg^2+^ , referred to as Gel **1**) and carboxymethyl chitosan hydrogel (CMCS, referred to as Gel **2**), are used to construct the micro‐gel ensembles. In the resultant micro‐gel ensembles, Gel **1** and Gel **2** contain ‐COOH and ‐NH_2_ separately, can respond to alkali and acid environment, respectively, and hence can release or adsorp H^+^ in the moist microenvironment of the wound, to achieve a dynamic acidic–alkaline regulation of wound pH (**Figure** **1**a). The two hydrogels could be assembled into various macrostructures (e.g., linear, planar, and 3D micro‐gel ensembles) via a microfluidic assembly technique, to cater the specific wound surface morphology (Figure 1b). Upon the treatment of wounds with micro‐gel ensembles, Gel **1** will release more H^+^ than the adsorption of Gel **2** at the initial stages (i.e., hemostasis and inflammation phases) so lowering the pH of wound surface. Later on the swelling of Gel **2** would expose more and more ‐NH_2_ to compromise the H^+^, resulting in a higher pH for the later hyperplasia and remodeling phases (Figure 1c,d). We also chelate Mg^2+^ to poly (HPA‐*co*‐AA) in Gel **1** as Mg^2+^ can effectively activate the F‐actin protein of adipocyte under acidic conditions, the differentiation and proliferation of which is very important at the initial stage of wound healing. Then we carry out in in vitro and in vivo experiments to verify that micro‐gel ensembles can effectively promote the formation of new blood vessels, the proliferation and migration of fibroblasts, and the polarization of macrophages through the adjustment of wound pH, and ultimately accelerate wound healing. As such, we design new micro‐gel ensembles allowing pH regulation over the four healing phases to achieve accelerated healing of chronic wound, which might provide a practical strategy for wound treatment and guide the development of the next generation of skin wound healing materials.

### 1.1 Preparation and Characteristics of Micro‐Gel Ensembles

**Figure 2:**
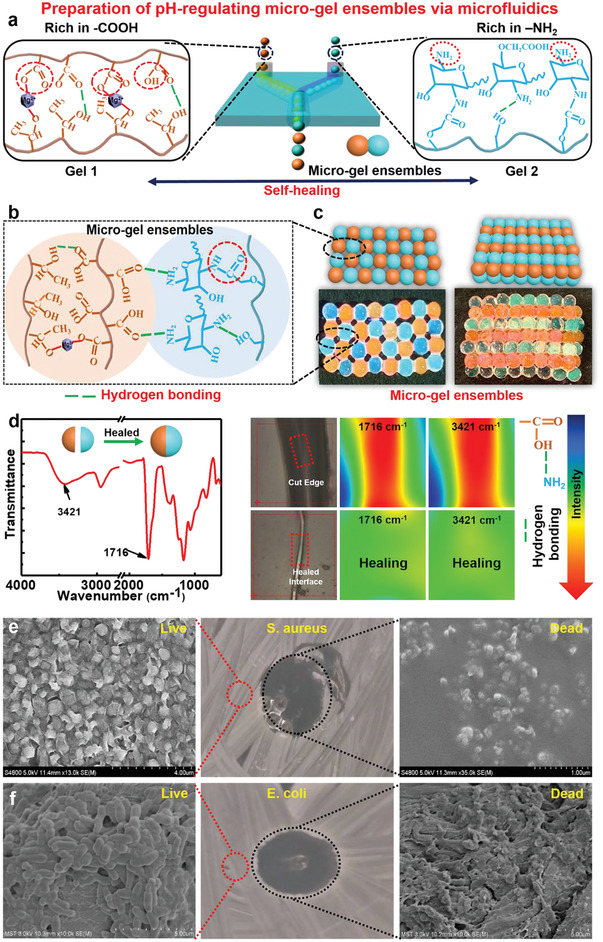
Preparation and characteristics of micro‐gel ensembles. a) Schematic illustration of preparing of micro‐gel ensembles via microfluidic technique. b) Schematic illustration of the molecular structure of micro‐gel ensembles. c) Construction of planar and 3D ordered assemblies by using Gel **1** and Gel **2** microbeads as building blocks via microfluidic assembly technique. d) IR spectrum, optical images, of Gel **1** and Gel **2** before and after self‐healing. SEM image of live/dead bacterial survival assay of e) E. coli, and f) S. aureus after in contact with the micro‐gel ensembles. *n* = 5 for *E. coli* and *S. aureus*.

We employed a microfluidic assembly technique to construct micro‐gel ensembles consisting of two biocompatible hydrogels of Gel **1 (**orange, rhodamine B staining) and Gel **2** (blue, fluorescein staining) (**Figure** **2**a). Initially, two types of soft uniform spherical gel microbeads of Gel **1** and Gel **2** were fabricated via a controllable and continuous microfluidic technique (Figure S1, Supporting Information). The mixture of HPA, AA, and Mg^2+^ was treated as discontinuous phase in a continuous microfluidic channel to form Gel **1** microbeads, where a UV beam was equipped to photopolymerize the monomers, and Mg^2+^ was bound to the carboxyl group (‐COOH) of the hydrogel through metal chelation (Figures S1–S3, Supporting Information). Gel **2** microbeads were constructed with microfluidic treatment of CMCS gels which were formed via chemical cross‐linking between the ‐NH_2_ and ‐COOH groups in CMCS to form amide bonds (Figures S1 and S4, Supporting Information).^[^ ^30^ ^]^ In the infrared (IR) spectrum of Gel **2** (Figure S5, Supporting Information), the absorption peak at 2925 cm^−1^ is assigned to the ‐CH_2_ stretching vibration, and the stretching vibrations of —NH, C═O, and C—N at 3419, 1615, and 1308 cm^−1^, respectively, indicate the formation of amide bonds. By adjusting the internal and external flow rates of the microfluidic chip, Gel **1** and Gel **2** microbeads with controllable and uniform sizes were fabricated (Figure S6 and Table S1, Supporting Information). Subsequently, based on the robust intermolecular hydrogen bonds between Gel **1** (containing ‐COOH) and Gel **2** (containing ‐NH_2_) (Figure 2b; Figure S7, Supporting Information), multi‐modal (e.g., linear, planar, and 3D) micro‐gel ensembles were fabricated from the microfluidic assembly of Gel **1** and Gel **2** (Figures 1b and 2c). The flexibility on macrostructures of micro‐gel ensembles was realized by simply employing specific microfluidic chips and channels, and hence micro‐gel ensembles with diverse macrostructures could be easily constructed to well fit the specific wound surface in wound treatments.

The micro‐gel ensembles made up of Gel **1** and Gel **2** microbeads are stable enough to remain shape persistent, owing to their good mechanical strength and self‐healing ability. As shown in Figure S8, Supporting Information, diverse macrostructures of micro‐gel ensembles are shape‐persistent and free‐standing when suspended vertically. To evaluate the self‐healing capacity between Gel **1** and Gel **2**, we split a Gel **1** microbead and a Gel **2** microbead mechanically in half, respectively, and then put one half of Gel **1** microbead and the other half of Gel **2** microbead in contact at room temperature (Figure 2d). After half an hour, the two halves fused autonomously, to afford an integrated sample of Gel **1** and Gel **2**. We employed microscopic infrared spectroscopy to investigate chemical changes from the cut edge to healed interface (Figure 2d). The IR imaging feature indicates that abundant dangling —C═O (1716 cm^−1^) and —NH_2_/—OH (3421 cm^−1^) groups distribute along the freshly cut edges, and then gradually decrease upon healing to give uniform distribution over the merged area. The results demonstrate that hydrogen bonds between Gel **1** (containing ‐COOH) and Gel **2** (containing ‐NH_2_) allow strong adhesion across the interface, thereby enabling the formation of an integrated microbead of Gel **1** and Gel **2**. Furthermore, both Gel **1** and **2** exhibit intrinsic self‐healing behavior without the need of inputting external stimuli (Figures S9 and S10, Supporting Information). We propose that the intermolecular hydrogen bonding between ‐OH of HPA and the ‐COOH of the AA enables strong adhesion across the rupture interface to realize self‐healing. Similarly, Gel **2** possesses excellent self‐healing properties probably owing to the formation of reversible amide bond between the ‐COOH (≈1640 cm^−1^) and ‐NH_2_ (≈3411 cm^−1^) groups as well as hydrogen bonding in Gel **2** (Figure S10, Supporting Information).^[^ ^34^ ^]^ Besides, the corresponding stress–strain curves show that self‐healed Gel **1** has good mechanical performance comparable with that of the normal sample (Figure S11, Supporting Information), and the healed sample show elongation at break over 400% (Figure S12 and Table S2, Supporting Information). By adjusting the mass ratio of HPA and AA monomers in Gel **1**, a healing efficiency of 92% could be achieved when HPA:AA = 6:4 (Figure S13, Supporting Information). The results suggest that micro‐gel ensembles are shape‐persistent and could self‐heal rapidly even when damaged, which could prevent or slow down mechanical damage to sustain normal peristalsis during wound healing treatments.

The as‐prepared micro‐gel ensembles have good swelling capacity. The swelling ratio of Gel **1** is 364% and that of Gel **2** is 302% (Figures S14 and S15 and Tables S3 and S4, Supporting Information). The high swelling ratio of the hydrogels is due to their porous and fluffy structure (Figure S16, Supporting Information). The swelling test results reveal that the micro‐gel ensembles have good‐to‐excellent exudate absorption capacity, and could effectively remove exudates from the wound while maintain a moist microenvironment.^[^ ^35^ ^]^

We further investigated the antibacterial capabilities of micro‐gel ensembles against *Staphylococcus aureus* and *Escherichia coli*. The scanning electron microscopy (SEM) images reveal the appearance of a bacteriostatic ring around the micro‐gel ensembles, and bacterial proliferation could be significantly inhibited by micro‐gel ensembles (Figure 2e,f). Gel **2** in the micro‐gel ensembles could absorb H^+^ from the wound microenvironment, transforming into NH_3_^+^_,_ which could eliminate and inhibit the proliferation of the bacteria (Figure S17, Supporting Information).^[^ ^36^ ^]^ Therefore, the micro‐gel ensembles have excellent antibacterial capabilities.

The vitro cytocompatibility of Gel **1**, Gel **2** and micro‐gel ensembles was also evaluated. The Live/Dead staining experiments were carried out by cell culture of L929 fibroblasts on micro‐gel ensembles. As shown in Figure S18, Supporting Information, the micro‐gel ensembles could maintain 90% cell viability after culturing the cells for 3 days, indicating the micro‐gel ensembles have good cytocompatibility and low cytotoxicity.

### 1.2 pH Regulation

**Figure 3:**
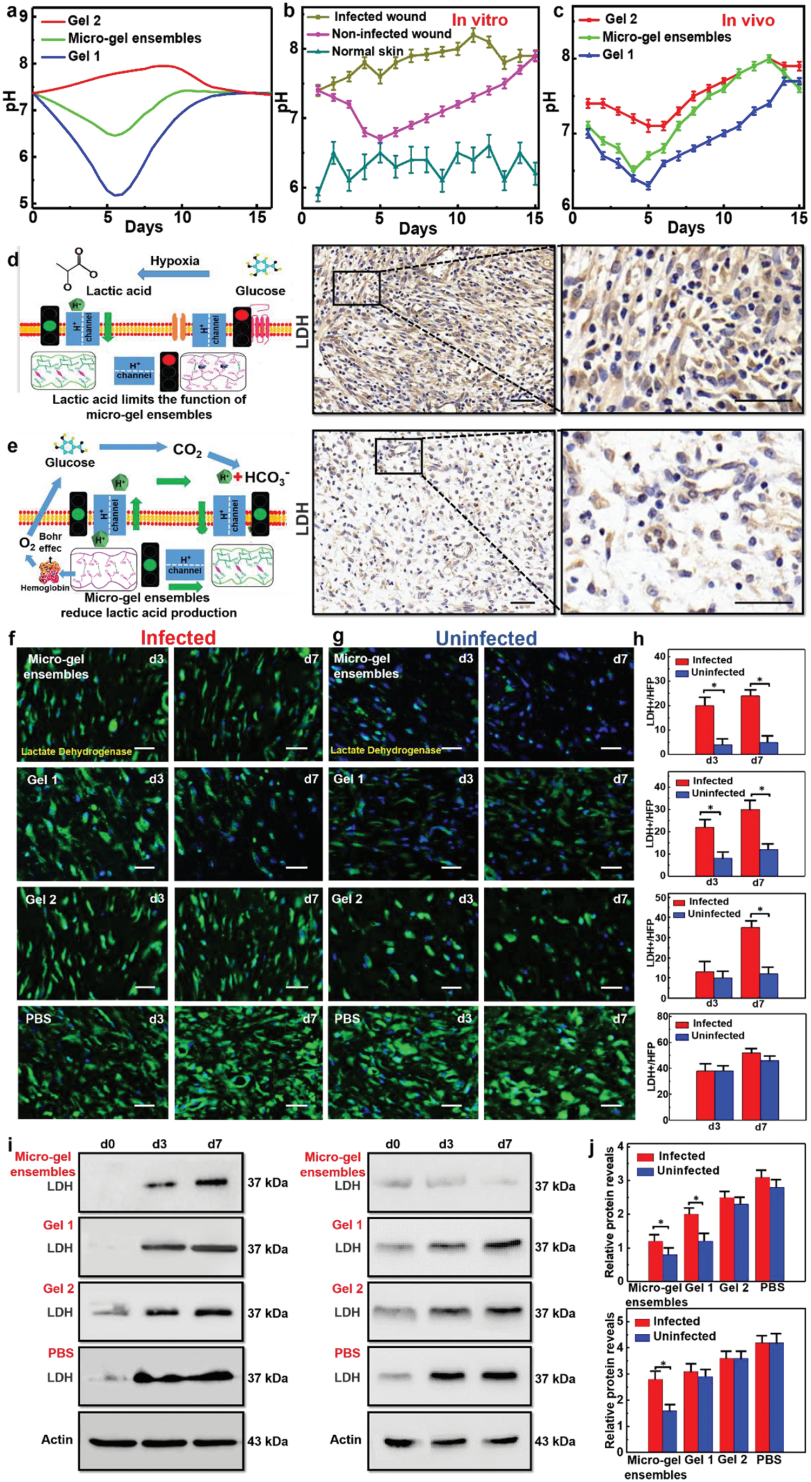
pH regulation of micro‐gel ensembles. a) The curves of the in vivo pH values of infected wounds, non‐infected wounds, and normal skin wounds with healing time. b) The curves of the in vitro pH values of Gel **1**, micro‐gel ensembles, and Gel **2** with time. Bars represent standard error, *n* = 5. c) The curves of the in vivo wound pH values of Gel **1**, micro‐gel ensembles, and Gel **2** with healing time. Bars represent standard error, *n* = 5. d) Schematic illustration of lactic acid affects pH regulation, which due to insufficient oxygen supply. e) The principle of micro‐gel ensembles to reduce lactic acid and achieve accurate pH control. Scale bars of (d) and (e) are 50 µm. f) The lactic acid (green) fluorescence staining of the granulation tissue on the third and seventh day of infected *S. aureus*. g) The above experimental results of uninfected group. Scale bars of (f) and (g) are 20 µm. *n* = 5 per group. h) The changes of lactate dehydrogenase (LDH) content at different times. Data were shown as mean values ± SD. Bars represent standard error, *n* = 5 per group. *p < 0.05. i) The expression levels of granulation tissue in different groups as detected by Western blot analysis (WB). j) WB gray values of granulation tissue after treating wounds with different materials. Data were shown as mean values ± SD. Bars represent standard error, *n* = 5 per group. *p < 0.05.

We investigated the controllable pH regulation capacity of micro‐gel ensembles on wounds. Firstly, we characterized the pH changes of infected wounds, non‐infected wounds, and normal skin over 15 days. As shown in **Figure** **3**a, the pH value of normal skin oscillates in the range 5.7–6.5, while the infected wounds have continuous alkaline environment. Whereas, during the non‐infected wound healing process, the pH first moves from alkaline (7.4) to acidic (6.7) and then back to alkaline (7.9). The results indicate a regulatable pH (i.e., alkaline–acidic–alkaline) environment is favorable for the wound healing.

Subsequently, in order to verify the pH regulation ability of micro‐gel ensembles, we first simulated the wound microenvironment through an in vitro chip, and found that when the in vitro pH value changes from 4 to 10, the micro‐gel ensembles can quickly and efficiently adjust the pH value of different chips to the range of 5–7 (Figure S19, Supporting Information). Next, we put three different gels (Gel **1**, Gel **2,** and micro‐gel ensembles) into the chip and extended the monitoring time to 15 days, proving that micro‐gel ensembles have the continuous pH regulation function (Figure 3b). Moreover, to find the right proportion (Gel **1**:Gel **2**) of micro‐gel ensembles, we used gel dressings containing different amounts of Gel **1** and **2** microbeads manufactured by adjusting the two‐phase flow rate of the Y‐type microfluidic chip, and detected the H^+^ concentration (Figure S20 and Table S5, Supporting Information). Remarkably, an increase in the amount of Gel **2** beads does not significantly affect the concentration of H^+^. On the contrary, as the number of Gel **1** microbeads increases, the H^+^ concentration of micro‐gel ensembles remains between the values of Gel **1** and Gel **2**.

Afterward, we applied the three types of gel microbeads (Gel **1**, Gel **2**, and micro‐gel ensembles) to the wound and recorded the daily pH value in vivo (Figure 3c). We found that the wound pH variation in all three groups shows similar trend which is decreased first and then increased. However, the pH for the wound treated with Gel **2** is always located in the alkaline range. Whereas, both the wounds treated with Gel **1** and with micro‐gel ensembles (Gel **1**:Gel **2** = 1:1) have regulatable alkaline–acidic–alkaline pH environments. Particularly, micro‐gel ensembles could regulate the pH completely in line with that for the non‐infected wound healing process, but with accelerated healing process (Figure 3a,c). This feature suggests that micro‐gel ensembles not only enable to change the wound pH, but also adjust the pH to an appropriate value. During the early hemostasis and inflammation stages of skin healing, the micro‐gel ensembles maintain the wound in a low‐pH microenvironment which could inhibit bacterial infection and promote vascular regeneration; during the later hyperplasia and remodeling stages, the micro‐gel ensembles regulate the pH to contribute an alkaline wound microenvironment which could promote cell proliferation and encourage skin remodeling. Thus, the micro‐gel ensembles are effective in regulating the pH of the wound surface, which is meaningful for promoting skin healing.

The above in vitro and in vivo experiments show that micro‐gel ensembles could precisely regulate pH value. Nonetheless, the question of why micro‐gel ensembles can precisely control the pH value of new granulation tissue in wounds still needs to be addressed. First, the lactate produced by newly generated tissue, due to insufficient oxygen supply, has the greatest impact on functionality. The lactic acid content is very high in the hyperplasia of wound healing, indicating that the hypoxia of the cells is serious (Figure 3d).^[^ ^37^ ^]^ Before forming neovascular network for the wound, the granulation tissue is always in a hypoxic state, resulting in large amounts of lactic acid. However, lactic acid has strong H^+^ desorption potential, which limits the exchange of H^+^ inside and outside the tissue, resulting in a localized low‐pH environment that is difficult to change.^[^ ^38^, ^39^ ^]^ Therefore, in order to verify the efficacy of micro‐gel ensembles, we compared the lactic acid production in different treatment groups, that is, the less lactic acid production, the less disturbance factors of wound pH value, and the more efficient micro‐gel ensembles regulation (Figure 3e).

We established four experimental groups (micro‐gel ensembles, Gel **1**, Gel **2**, and PBS) to treat different skin wounds located on the backs of rats which were infected with *S. aureus* and were without bacterial infection, respectively (Figure 3f,g). The lactic acid expression levels in the tissues after 0, 3, and 7 days were detected by Western blot analysis (Figure 3h). We then compared the effects of lactic acid production on different wound surfaces and different time of wound surfaces. The results of fluorescence staining showed that the wound microenvironment was adjusted to acid by micro‐gel ensembles, and the oxygen released by hemoglobin increased (Bohr effect), so the hypoxia of tissue was relieved. Reducing the production of lactic acid could conduce to ensure the precise pH regulation of wound microenvironment by micro‐gel ensembles. Further verification of the lactic acid in the tissues after 0, 3, and 7 days was accomplished by Western blot analysis (Figure 3i). According to the results shown by the grayscale values, the micro‐gel ensembles significantly reduced the production of lactic acid in new granulation tissue (Figure 3j). Whereas, although Gel **1** microbeads locally reduced the production of lactic acid in the new granulation tissue, the density of new cells was significantly lower than that of other groups, and the continuous acidic environment on the surface could inhibit the proliferation of cells. And alkaline environment created by Gel **2** aggravated the anoxic environment of the new wound tissue, resulting in a large amount of lactic acid accumulation. The results prove that the acid environment afforded by Gel **1** can reduce the production of lactic acid, but it will limit the proliferation of cells. On the contrary, the alkaline environment constructed by Gel **2** will aggravate the hypoxia of new tissue. It is gratifying that micro‐gel ensembles composed of Gel **1** and Gel **2** enable the mutual transformation of acid‐base environment and fully realize the transformation.

### 1.3 Accelerated Skin Healing

**Figure 4:**
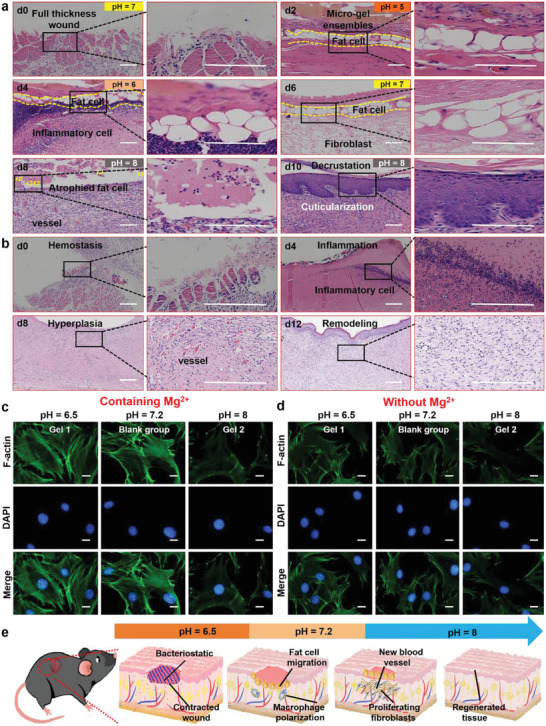
In vitro and in vivo validation of adipocytes activation and fibroblast proliferation. a) H&E fluorescence staining images of new tissue at different stages after micro‐gel ensembles treatment of full‐thickness skin defects. b) H&E fluorescence staining images of the four healing stages (hemostasis, inflammation, hyperpiasia, remolding) of chronic wounds over time. The scale bars of (a) and (b) are 200 µm, *n* = 5 per group. Fluorescence staining images of gel materials c) with Mg^2+^ and d) without Mg^2+^ where the results showed that gel materials containing Mg^2+^ effectively activated the F‐actin of adipocytes in an acidic environment. The scale bars of (c) and (d) are 5 µm, *n* = 24. e) Schematic illustration of wound healing process (early protection, macrophage polarization, new blood formation, and tissue regeneration).

After verifying the precise regulation of wound pH by micro‐gel ensembles, we performed histological analysis to find out how micro‐gel ensembles interact with new wound tissue at different times. We discovered that a mass of adipocytes appeared around the micro‐gel ensembles after 2 days of treatment. Moreover, adipocytes were evenly distributed along the wound surface, in the form of a protective wall along the wound. And inflammatory cells appeared on the medial side on day 4. However, almost no inflammatory cells were present, and fibroblasts began to appear under the adipocyte wall on day 6. Subsequently, the adipocytes started undergoing apoptosis, and the fibroblasts on the wound began to proliferate on day 8. Finally, cuticles began to form on the wound surface, indicating skin healing on day 10 (**Figure** **4**a). This wound healing process not only involves the temporal and spatial synchronization of multiple cell types, but also shortens the healing time compared with normal wound healing process (hemostasis, inflammation, hyperplasia, and remodeling) (Figure 4b). Moreover, this feature of wound healing involving adipocyte proliferation and migration in this case are consistent with recent research in Drosophila that uses the peristalsis of adipocytes to treat wounds.^[^ ^40^, ^41^ ^]^ The results show micro‐gel ensembles with wound pH regulation ability enable accelerated skin healing.

Further investigation revealed that micro‐gel ensembles allow the activation of adipocytes and accelerated proliferation of fibroblasts in the wound, thereby promoting skin healing. To analyze the induce of adipocytes participating in wound healing, we established three sets of experiments containing Mg^2+^ (Gel **1** group, a blank group, and a Gel **2** group) and three sets of experiments without Mg^2+^ (poly (HPA‐*co*‐AA) group, a blank group, and a Gel **2** group) (Figure 4c,d). The activation of F‐actin was analyzed by fluorescence staining, and the Gel **1** group exhibited the best activation effect on F‐actin in an acidic environment compared to the other three groups. Thus, adipocyte activation occurs best under acidic conditions in the presence of Mg^2+^. A previous study found that Mg^2+^ can activate actin and promote cell proliferation and migration.^[^ ^42^ ^]^ However, it is difficult to activate adipocytes under alkaline conditions (Figure 4c,d, right), mainly because it is difficult for Mg^2+^ to persist in alkaline environments, resulting in limited F‐actin activation. Moreover, the skin healing rate also depends on the proliferation rate of fibroblasts,^[^ ^43^ ^]^ with particular emphasis on the fact that alkaline environments are more conducive to fibroblast proliferation.^[^ ^44^, ^45^ ^]^ Therefore, we conducted a systematic study on the proliferation of fibroblasts through fluorescence staining experiments. The results show that Gel **2** provides an alkalinity pH microenvironment for fibroblast proliferation. However, the acidic microenvironment of Gel **1** inhibits the proliferation of fibroblasts (Figure S21, Supporting Information). The combination of Gel **1** and Gel **2** into micro‐gel ensembles enables the availability of collective functions of Gel **1** and Gel **2** whilst possessing pH regulation ability, resulting in the activation of adipocytes, accelerated proliferation of fibroblasts and thus promoted skin healing.

Based on the above experimental results, we summarize the skin healing process treated with micro‐gel ensembles, along with changes in wound pH (Figure 4e; Figure S22, Supporting Information). Initially, when the skin structure is destroyed, the treatment with micro‐gel ensembles allows the pH values of the wound changing from 7.4 to around 6.5. In this low‐pH microenvironment, adipocytes are vigorously activated by Mg^2+^ present in the micro‐gel ensembles, and then migrate to the wound site, forming an adipocyte wall to protect the wound. Due to the adipocyte wall maintaining the integrity of the epidermis in the early stages, pathogenic infection of the wound is reduced.^[^ ^46^ ^]^ During the inflammatory stage, the pH values of wound increases from about 6.5 to ≈7.2 and adipocytes work with macrophages to eliminate necrotic tissue and cells. Meanwhile, adipocytes stimulate the expression of HIF‐1*α*, which in turn promotes the synthesis of ECM by fibroblasts, allowing for macrophage polarization and the formation of new blood vessels.^[^ ^47^ ^]^ Subsequently, the pH values of the wound change from 7.2 to around 8.0, and adipocytes begin to supply nutrients to the surrounding new granulation tissue, thus accelerating cell proliferation. Finally, the adipocytes atrophy in the scabs, which protect the fresh wound tissue. These findings provide new insights into the skin healing strategies, and are significant for treating the full thickness of the skin. Also, this solution may provide an easy and cost‐effective method for repairing large wound areas.

### 1.4 Evaluation of Final Healing Effects

**Figure 5:**
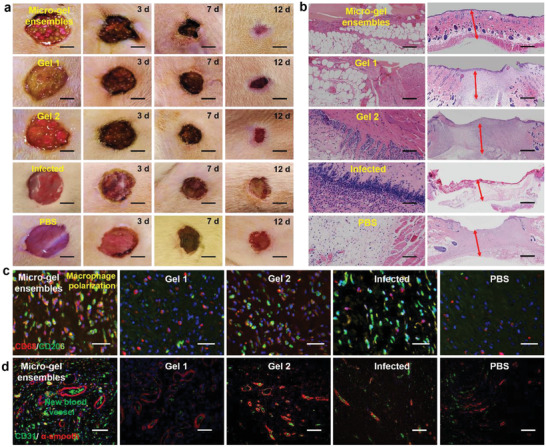
Four processes (wound healing area, granulation growth thickness, macrophage polarization, and neovascularization regeneration) of skin formation. a) Digital photographs of representative skin wound healing processes in rats treated with micro‐gel ensembles, Gel **1**, Gel **2**, infected, and PBS, respectively. The scale bars are 1 cm, *n* = 7 per group. b) Microscopy images of granulation growth of micro‐gel ensembles, Gel **1**, Gel **2**, infected, and PBS groups by H&E. The scale bars are 200 µm, *n* = 7 per group. Double immunofluorescence staining images of c) macrophages, and d) new blood vessels in the micro‐gel ensembles, Gel **1**, Gel **2**, infected, and PBS groups. The scale bars of (c) and (d) are 50 µm, *n* = 7 per group.

We further systematically investigated the vital applications of micro‐gel ensembles in skin healing and skin regeneration by quantifying the size of the wound healing area, the polarization of macrophages, and the formation of new blood vessels (**Figure** **5**). We first created five groups (micro‐gel ensembles, Gel **1**, Gel **2**, PBS, and normal hydrogel groups) of experiments to treat *S. aureus*‐infected wounds, using a group of uninfected wounds as the control. The wound treated with micro‐gel ensembles was almost completely healed after 12 days, and the healing efficiency was 93%, while the healing efficiency of the Gel **1** group was 81%, the Gel **2** group was 65%, the PBS group was 66%, and the normal hydrogel group was 68% (Figure 5a; Figure S23a, Supporting Information). The results revealed that the micro‐gel ensembles could accelerate skin wound healing. Additionally, we performed histological analysis of the healed skin tissue. Figure 5b shows that the micro‐gel ensembles group not only promotes the proliferation and migration of adipocytes, but also enhances the thickness of granulation growth. Although the Gel **1** group could also promote the proliferation and migration of adipocytes, the healing rate was slower. Besides, the other four groups (uninfected, PBS, Gel **2**, and normal hydrogel groups) have no adipocytes involved (Figure 5b; Figure S23b, Supporting Information), and the Gel **2** group has tissue hyperplasia due to the excessive proliferation of fibroblasts. The results indicate the micro‐gel ensembles realize accelerated scarless skin healing.

Finally, we observed numerous M2‐type macrophages (red) in the wound granulation tissue with an adipocyte layer via double immunofluorescence staining (Figure 5c), and the number of neovascularization for the group treated by micro‐gel ensembles was significantly more than other three groups (Figure 5d). This indicates that adipocytes play an irreplaceable role in promoting macrophages to M2‐type polarization. Moreover, numerous studies have shown that the polarization of macrophages to M2 not only promotes angiogenesis but also increases the formation of collagen fibers and promotes tissue repair and remodeling.^[^ ^6^, ^48^ ^]^ Combined results from Figure 5c,d further verify that the polarization of macrophages and the formation of new blood vessels accelerate skin healing.

## 2 Conclusion

In conclusion, we developed micro‐gel ensembles that could precisely regulate wound pH value to facilitate the wound healing process. The micro‐gel ensembles composed of Gel **1** and Gel **2** microbeads were prepared via microfluidic assembly, to show good mechanical strength, self‐healing ability, swelling capacity, and antibacterial capability. Importantly, in vitro and in vivo experiments demonstrated that the micro‐gel ensembles allow the continuous regulation on the wound pH over the four wound healing phases (hemostasis, inflammation, hyperplasia, and remodeling). During the hemostasis and inflammation stages, the micro‐gel ensembles maintain the wound microenvironment in a low pH to inhibit bacterial growth. Meanwhile, the acidic milieu is favorable for the stimulation of Mg^2+^ to activate the proliferation and migration of adipocytes, forming a wall to protect the wound. Then the micro‐gel ensembles regulate an optimal alkaline environment for hyperplasia and remodeling, in which proliferation and migration of fibroblasts accelerate skin healing. The micro‐gel ensembles could also promote collagen deposition, the polarization of macrophages, and the formation of new blood vessels, facilitating skin healing. In general, this work contributes a proof‐of‐concept approach to new wound healing materials accelerating the skin wound healing process, and might provide new insights into skin healing strategies for large‐area wound repairing.

## 3 Experimental Section

### Materials

Hydroxypropyl acrylate (HPA), acrylic acid (AA), glycerol, methyl silicone oil, carboxymethyl chitosans (CMCS), dimethyl sulfoxide (DMSO), *n*‐hexane，N,N‐dimethylformamide (DMF)，magnesium chloride (MgCl_2_) were purchased from Sinopharm Chemical Reagent Co (Shanghai, China). 1‐Ethyl‐3‐(3‐dimethylaminopropyl) carbodiimide hydrochloride (EDC), *N*‐hydroxysuccinimide (NHS), *N,N*‐methylenebisacrylamide (MBA), methanol, HCl solution (4 m), NaOH, rhodamine B, and fluorescein were supplied by Aldrich and used as received. All chemicals were used without any further purification. Ultrapure water (18 MΩ cm) from a Milli‐Q water system was used throughout the experiments.

### Preparation of Gel **1** Microbeads by Microfluidic Technique

6 g HPA, 4 g AA, 0.033 g MBA, 0.5 g MgCl_2_, 0.5 g photoinitiator 2959 were dissolved in the 6.7 g DMF, and stirred vigorously in a 25 mL beaker to prepare Gel **1** precursor solution. Then, the precursor solution (internal discontinuous phase) was injected into a 15 mL syringe and attached to a Y‐type microfluidic chip, and the other phase was an external continuous phase methyl silicone oil to shear the precursor solution to form droplets. Finally, the droplets of precursor solution were irradiated with ultraviolet light for 30 s to initiate radical polymerization, yielding the hydrogel microbeads of Gel **1**. By adjusting the flow rates of internal/external phase, Gel **1** microbeads with different sizes were fabricated.

### Preparation of Gel **2** Microbeads by Microfluidic Technique

CMCS (3 g) and small amount of rhodamine B were dissolved in water of 97 g to prepare 3% CMCS solution at ambient temperature. After well mechanically stirring, filtering, and then degasing, the solution was injected into a 15 mL syringe for use as the discontinuous phase (internal phase), sheared by the continuous phase of tetramethyl silicone oil (external phase), to form CMCS droplets. Those CMCS droplets were collected by a dish containing 40 mL of DMSO solution of EDC (0.1 g) and NHS (0.06 g), where in situ cross‐linking occurred to form Gel **2** microbeads. Gel microbeads with different diameters were prepared by adjusting the two‐phase microfluidic flow rate.

### Preparation of Micro‐Gel Ensembles by Microfluidic Technique

A microfluidic system equipped with a Y‐type microfluidic chip was used for preparation of micro‐gel ensembles. Gel microbeads of Gel **1** and Gel **2** in tetramethyl silicone oil were introduced into the Y‐type chip separately, which met at the junction. By adjusting the two‐phase microfluidic flow rates, alternating arrangements of two types of microbeads were achieved, ultimately forming multi‐modal (e.g., linear, planar, and 3D) micro‐gel ensembles through self‐assembly. All samples of micro‐gel ensembles for further use were washed by *n*‐hexane to remove tetramethyl silicone oil on the surface of gels, and then immersed in deionized water for 3 days to remove unreacted monomer and organic solvents by changing the water every day.

### Wound pH Detection

The wound conditions were observed and were grouped. A portable pH acidity meter glass plane was used to click to detect the pH of the wound before and after debridement, as well as the pH of infected wounds, non‐infected wounds, and normal skin around rat wounds.

### Measurements

The gel microbeads were also observed by an inverted fluorescence microscope (SFM‐30I, Shanghai), which measure the size of the gel microbeads. IR images were performed on a Thermo Scientific Nicolet In10 infrared microscope equipped with a liquid nitrogen cooled MCT detector (Thermo Electron Corporation, USA). IR microscopy data were collected using reflection mode. IR spectra were captured using an aperture size of 50 µm × 50 µm and were recorded over a range of 500–4000 cm^−1^. An analysis of the IR microscopy data was performed using OMNIC picta software (Thermo Electron Corporation, USA). The mechanical tensile stress–strain test was carried out by using a SANS CMT6203 testing machine at room temperature.

### In Vitro Biodegradation Experiment of Gel **2**

To study the degradation performance of Gel **2** in vitro, a certain quality of Gel **2** was weighed and then the Gel **2** sample was placed in a PBS solution containing lysozyme (10 mg mL^−1^) at 37 °C (Figure S24, Supporting Information). For statistical analysis, *n* = 3.

### Antibacterial Experiments

The *E. coli* and *S. aureus* were cultured on the lactose peptone dextrose agar medium for 48 h, and then were collected by PBS. The concentration of the bacteria suspension was diluted to 10^8^ per mL. Later 10 µL bacterial suspensions and microspheres were cultured with LB (Luria‐Bertani) agar for 24 h at 37 °C. For statistical analysis, *n* = 5 for *E. coli* and *S. aureus*.

The bacteria cell morphologies of different gel microspheres were examined using a SEM. Briefly, microbial cells were incubated with different gel microspheres for 24 h, after that the bacterial cells were mixed with 5% glutaraldehyde in 0.15 m sodium phosphate buffer (pH 7.4) for 1 h. Subsequently, Gel **1**, Gel **2**, and micro‐gel ensembles with bacterial cells were rinsed with 0.15 m sodium phosphate buffer and dehydrated through a graded ethanol series (25–100%). These gels were dried under nitrogen, and SEM images were acquired at an accelerating voltage of 10.0 kV and a working distance of 10.0 mm.

### Cell Culture

i) Gel **1** and Gel **2** were UV sterilized (30 min) before placing in ultralow adhesion 24‐well plates and the differentiated 3T3‐L1 cells (ATCC) was added. 0.5 mL EMEM medium containing 10% NBS was added and the samples were cultured at 37 °C with a humidified atmosphere of 5% CO_2_. ii) Gel **1** and Gel **2** were UV sterilized (30 min) before placing in ultralow adhesion 24‐well plates. 40 000 L929 fibroblasts were seeded per well. 0.5 mL EMEM medium containing 10% FBS was added and the samples were cultured at 37 °C with a humidified atmosphere of 5% CO_2_. All experiments were done in triplicate. Cell survival on the Gel **1** and Gel **2** were studied using a live–dead assay. At 72 h, the fat cells were stained with anti‐Actin antibody (abcam 179467), fibroblasts were stained with calcien AM and propidium iodide according to the manufacturers’ instructions. The viability of cells inside the hydrogels was observed under a Leica fluorescence microscope.

### Experiment on Wound Healing in Rats

SD rats (8 weeks old, 220–260 g) were anesthetized through intraperitoneal injections of a ketamine (50 mg kg^−1^ body weight) and xylazine (5 mg kg^−1^ body weight) mixture, then their backs were shaved. A rounded full‐thickness cutaneous wound (1.5 cm × 1.5 cm) area was created on the back of each rat, and then divided into five groups randomly. Four groups were infected with *S. aureus*. The four infected groups were treated with Gel **1**, Gel **2**, micro‐gel ensembles, and PBS respectively. Here, Gel **1**, Gel **2**, and micro‐gel ensembles with size of 1.5 cm × 1.5 cm were directly employed to cover the rat wound. Thereafter, the rats were individually housed in cages and allowed to heal for 12 days. The wounds were observed and the granulation tissues were excised on days 0, 3, 7, and 12. Each sample was divided into two pieces. One piece was immersed in neutral formaldehyde for further Western blots, histology and immunohistochemistry analysis, another was stored in liquid nitrogen for immunofluorescent staining. For statistical analysis, *n* = 7 per group.

### Western Blots, Histology and Immunofluorescence Staining

The frozen tissue samples of 0, 3, and 7 days were taken out for cleavage, electrophoretic, transmembrane, and staining of the samples according to the instructions for anti‐lactate dehydrogenase B/LDH‐B antibody (abcam 75 167) use. The granulation tissue samples were removed from the neutral formaldehyde, followed by dehydration and embedded in paraffin. Serially sections, 5 µm in thickness, were acquired by a microtome according to standard protocols, and were prepared for hematoxylin‐eosin and immunofluorescence staining. Fluorescent staining of lactate was carried out on 3‐day and 7‐day tissue sections according to the instructions for anti‐lactate dehydrogenase B/LDH‐B antibody use. For statistical analysis, *n* = 5 per group. Fluorescent staining of neovascularization in tissue sections according to instructions for primary antibodies against CD31 (KEYGEN, KGYM0118–7) and *α*‐smooth muscle actin (*α*‐SMA) (KEYGEN, KGYT5053‐6). The frozen samples were fixed in acetone, and then incubated with blocking buffer. According to the steps of immunofluorescence staining, the macrophages of tissue sections were stained with antibodies CD68 (Abcam, ab955) and CD206 (Santa Cruz, sc‐34577). For statistical analysis, *n* = 7 per group.

### Ethical Certification

This study was carried out according to the Recommendations of Guidelines for Clinical Trials by the Ethics Committee of Jinling Hospital. All animal experiments in this study were performed according to the principles of the Declaration of Helsinki, and were approved by the Animal Ethical Committee of Jinling Hospital (2018GKJDWLS‐03‐127).

### Statistical Analysis

Quantitative data were shown as mean ± standard deviation (SD). The significance between groups was assessed by unpaired Student's *t*‐test or one‐way analysis of variance. The differences were considered significant at *p* < 0.05 (*p < 0.05; **p < 0.01; ***p < 0.001). The graphs and statistical analysis were carried out using GraphPad Prism software, version 7 and OriginPro 2020 (Learning Edition). Histological examination was performed by capturing at least five fields per section and images were analyzed using Image J software.

## Conflict of Interest

The authors declare no conflict of interest.

## Supporting information

Supporting Information

## Data Availability

The data that support the findings of this study are available from the corresponding author upon reasonable request.
