# Supplementary material for: Micro‐Gel Ensembles for Accelerated Healing of Chronic Wound via pH Regulation
Source: Adv Sci (Weinh). 2022 May 21;9(22):2201254. doi: 10.1002/advs.202201254 (PMC9353480; doi:10.1002/advs.202201254)
Supplement: Supplementary file 1 — Supporting Information [file ADVS-9-2201254-s001.pdf]

ADVANCED SCIENCE Open Access

Supporting Information

for *Adv. Sci.*, DOI 10.1002/advs.202201254

Micro-Gel Ensembles for Accelerated Healing of Chronic Wound via pH Regulation

Tingting Cui, Jiafei Yu, Cai-Feng Wang, Su Chen*, Qing Li, Kun Guo, Renkun Qing, Gefei Wang* and Jianan Ren*

Supporting Information

Micro-Gel Ensembles for Accelerated Healing of Chronic Wound via pH Regulation

Tingting Cui, Jiafei Yu, Cai-Feng Wang, Su Chen*, Qing Li, Kun Guo, Renkun Qing, Gefei Wang*, Jianan Ren*

Dr. T. Cui, Prof. C.-F. Wang, Prof. S. Chen, Dr. Q. Li, R. Qing
State Key Laboratory of Materials-Oriented Chemical Engineering, College of Chemical Engineering, Jiangsu Key Laboratory of Fine Chemicals and Functional Polymer Materials, Nanjing Tech University, Nanjing 210009, P. R. China.
E-mail: chensu@njtech.edu.cn, caifengwang@njtech.edu.cn
J. Yu, K. Guo, Prof. G. Wang, Prof. J. Ren
Department of General Surgery, Jinling Hospital, Nanjing Medical University, Nanjing 210002, China.
E-mail: Gefei_W@163.com

Supporting Figures

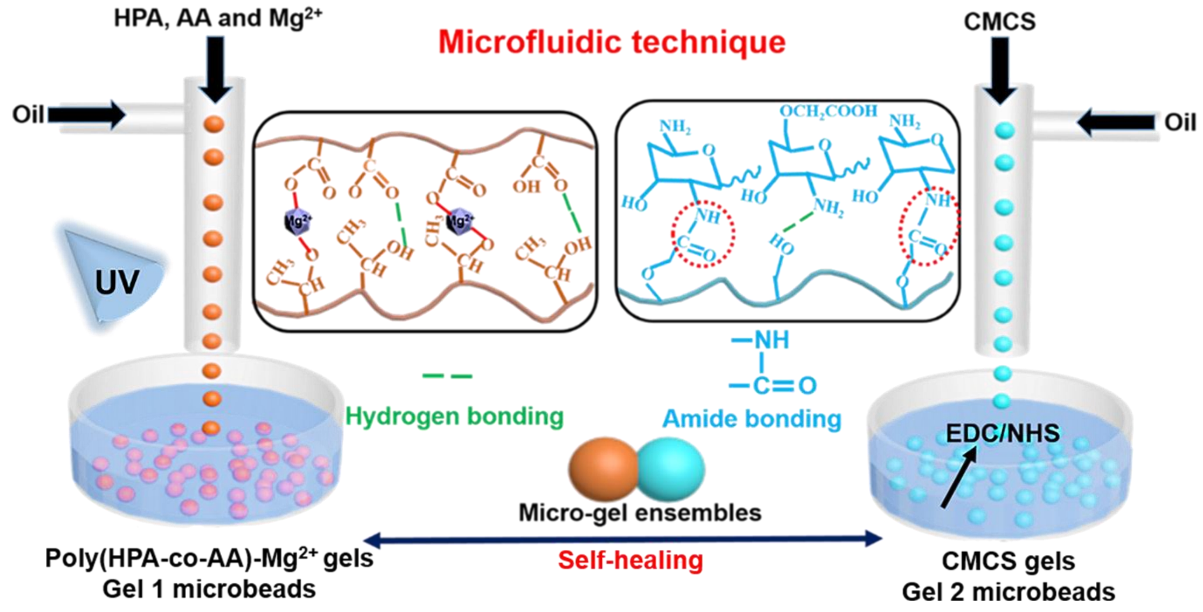

Figure S1. Preparation of Gel **1** and Gel **2** microbeads via microfluidic technique.

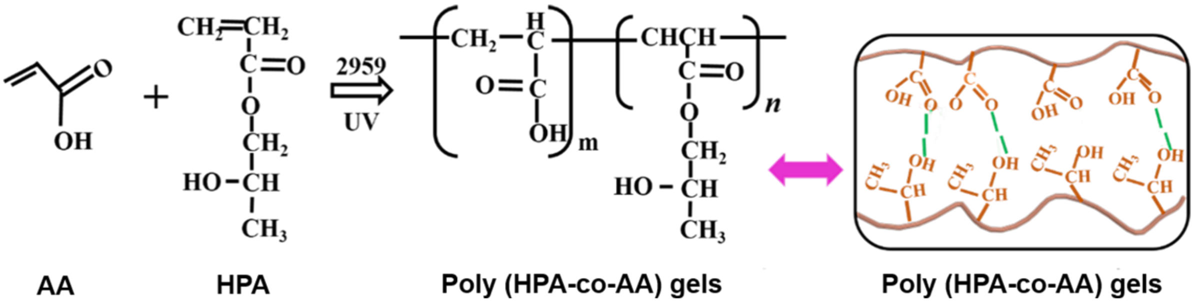

Figure S2. Schematic synthesis of poly (HPA-co-AA) gels.

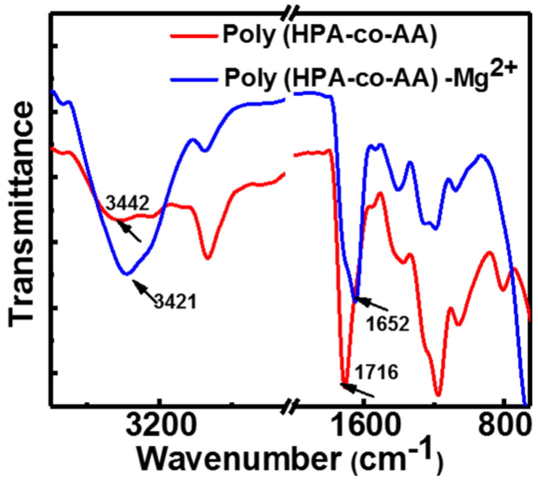

Figure S3. IR spectra of poly (HPA-co-AA) gel and poly (HPA-co-AA)-Mg^{2+} gel.

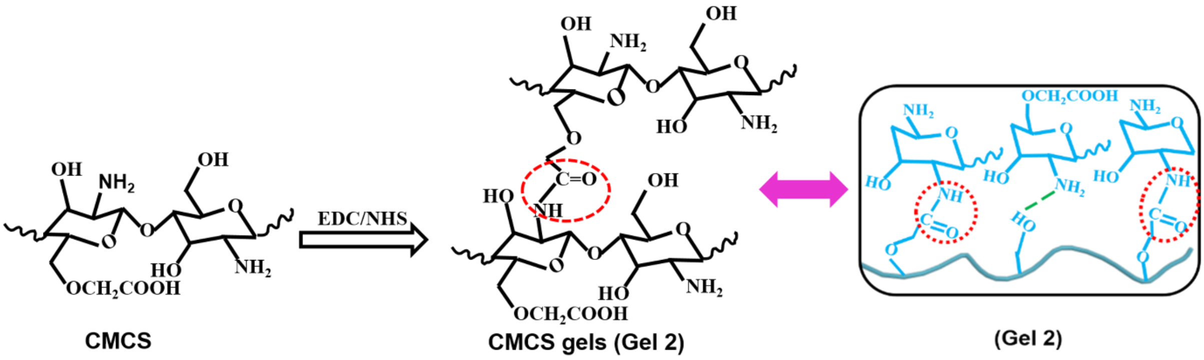

Figure S4. Schematic synthesis of CMCS gel (Gel **2)**.

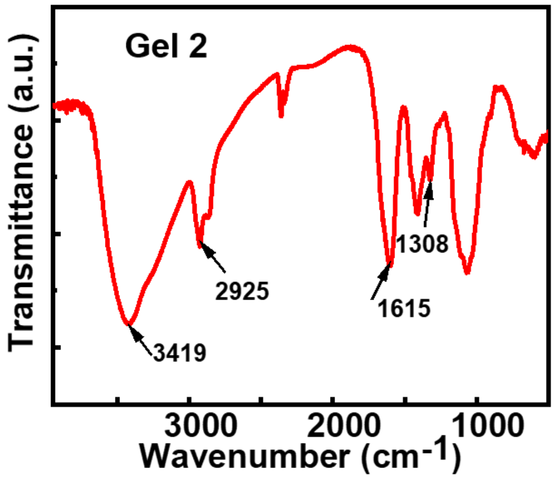

Figure S5. IR spectrum of Gel **2**.

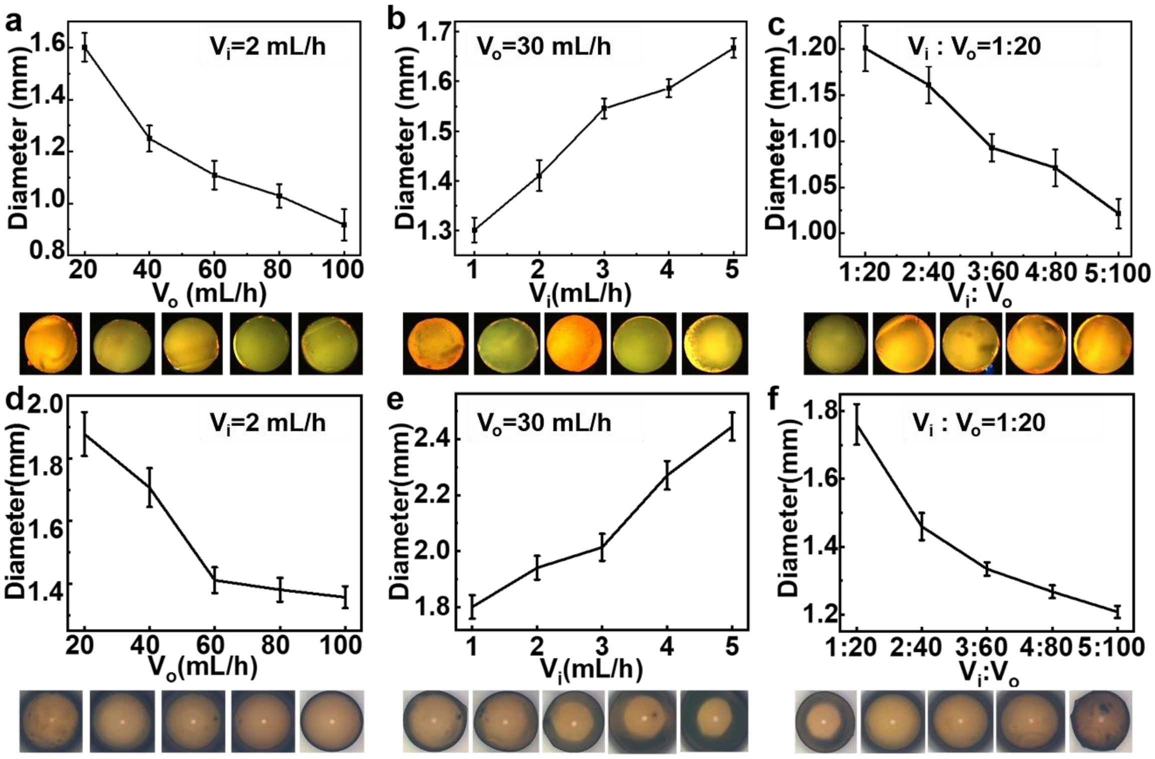

Figure S6. Dependence of microbead size on a,d) internal flow rate, b,e) external flow rate, and c,f) internal/external flow rate for a-c) Gel **1** microbeads and d-f) Gel **2** microbeads. Bars represent standard error, for statistical analysis in a-f, n = 4 and field of view (FOV) ≥4.

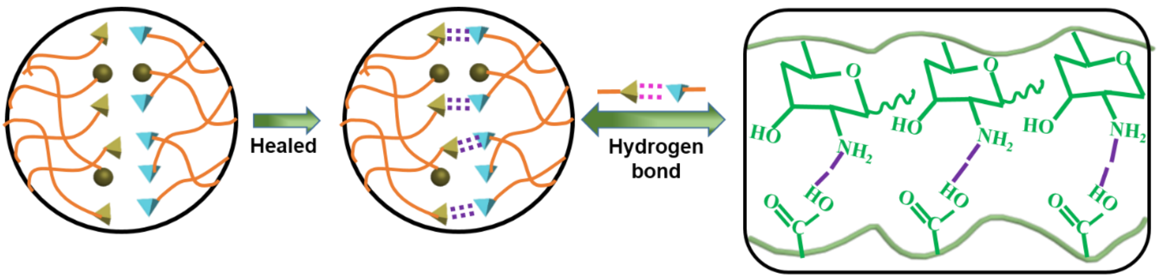

Figure S7. Schemaic representation of the healing process between Gel **1** and Gel **2** via the hydrogen interaction.

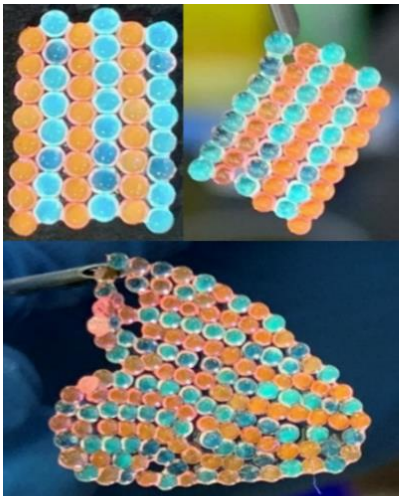

Figure S8. Photographs demonstrate that the micro-gel ensembles can remains shape persistent.

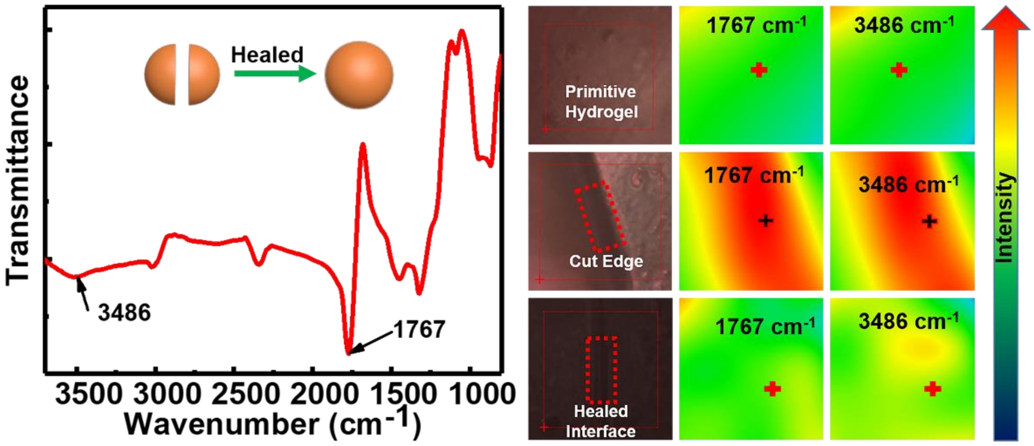

Figure S9. IR spectra, Optical images and IR images of Gel **1** before and after self-healing.

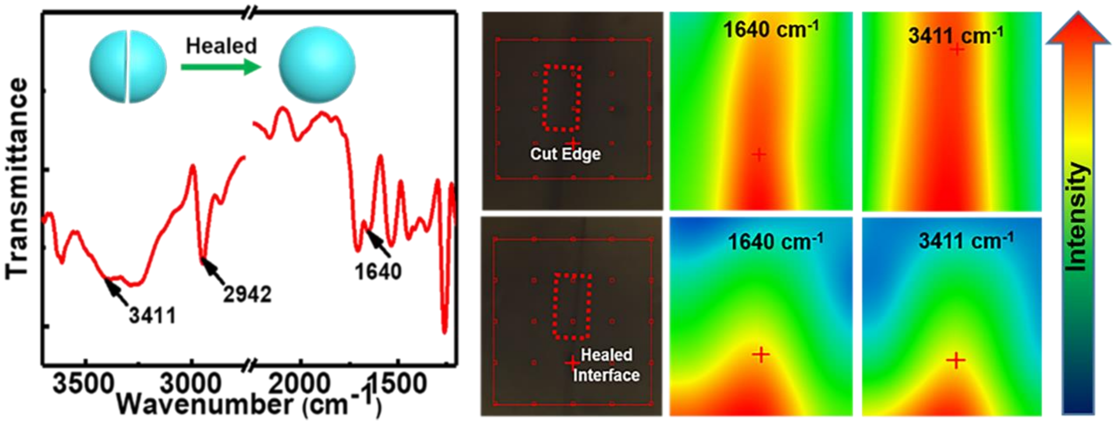

Figure S10. IR spectra, Optical images and IR images of Gel **2** before and after self-healing.

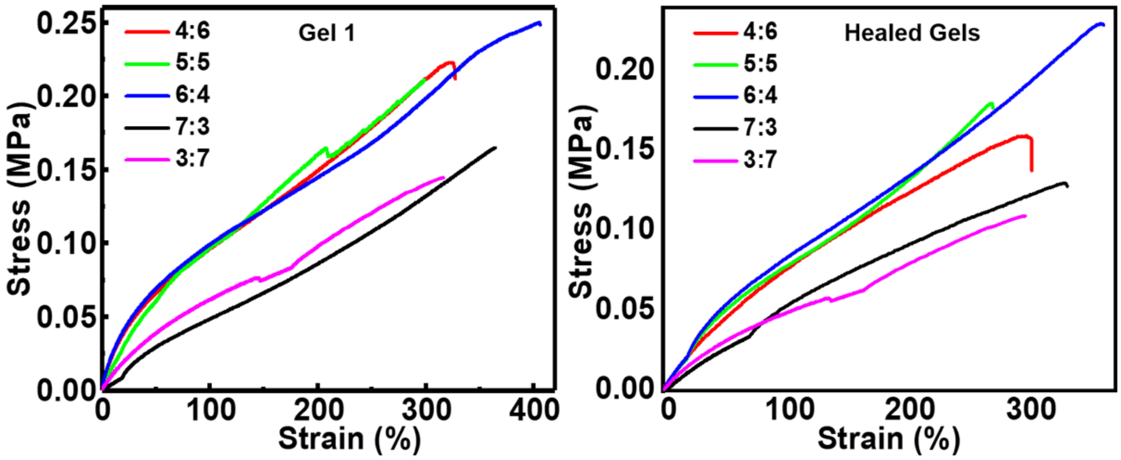

Figure S11. Stress-strain curves of Gel **1** and healed Gel **1** obtained from different mass ratios of HPA/AA) at a stretching rate of 0.2 mm/s. For statistical analysis, n = 3.

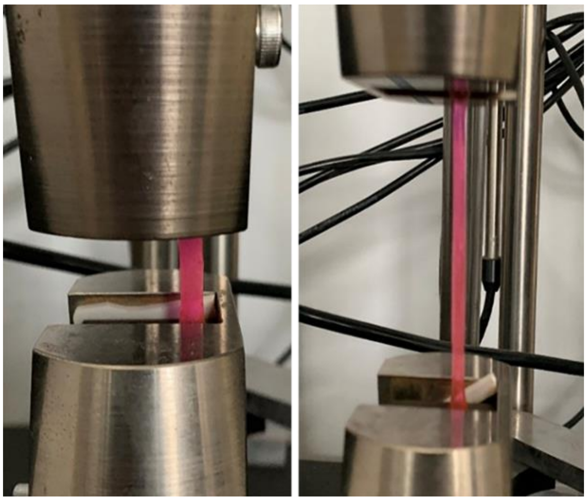

Figure S12. Photographs demonstrating the good mechanical properties of Gel **1.**

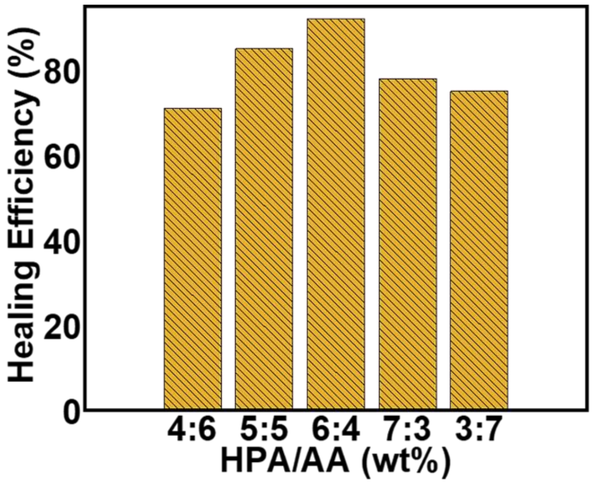

Figure S13. Self-healing efficiency of Gel **1** at different mass ratios (HPA:AA).

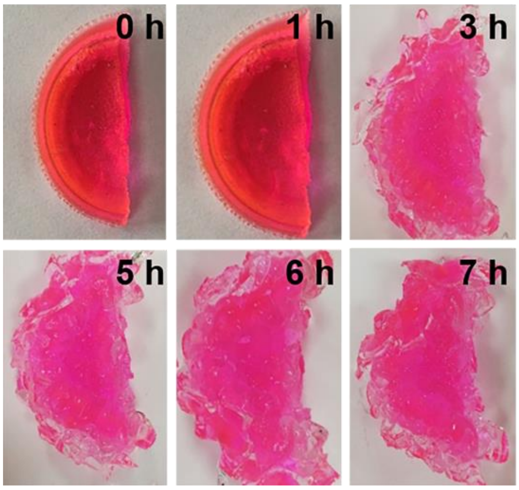

Figure S14. Swelling performance of Gel **1** in different time periods.

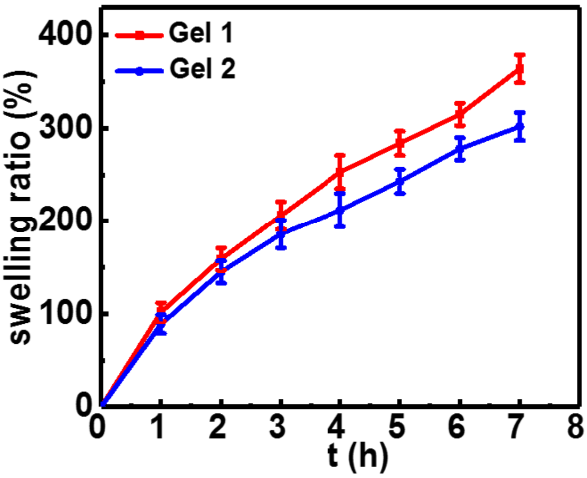

Figure S15. Swelling performance curves of Gel **1** and Gel **2**. Bars represent standard error, for statistical analysis, n = 3.

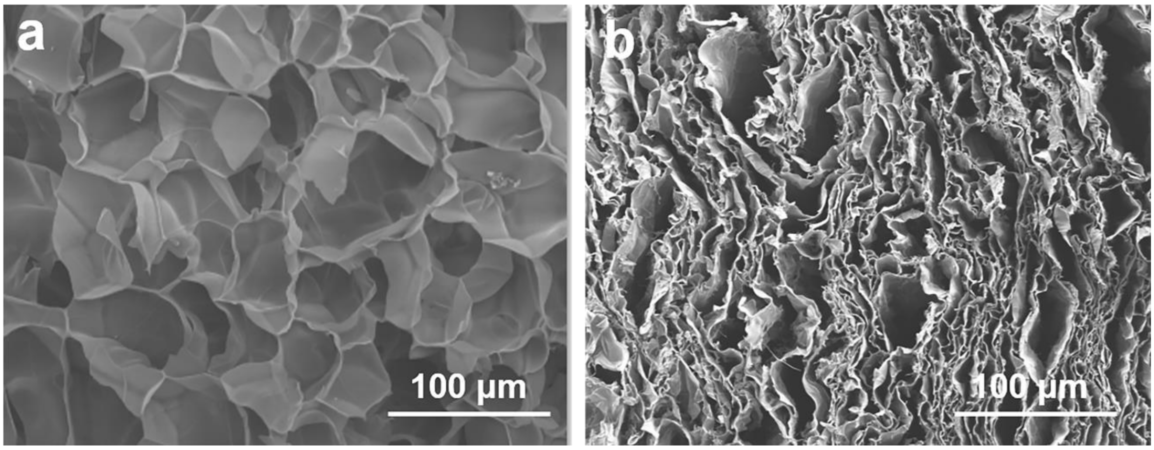

Figure S16. SEM images of a) Gel **1** and b) Gel **2**.

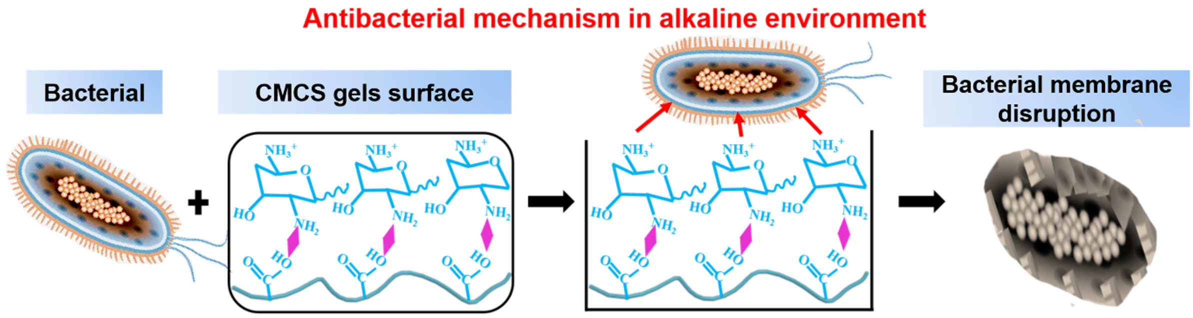

Figure S17. Schematic illustration of the antibacterial of micro-gel ensembles.

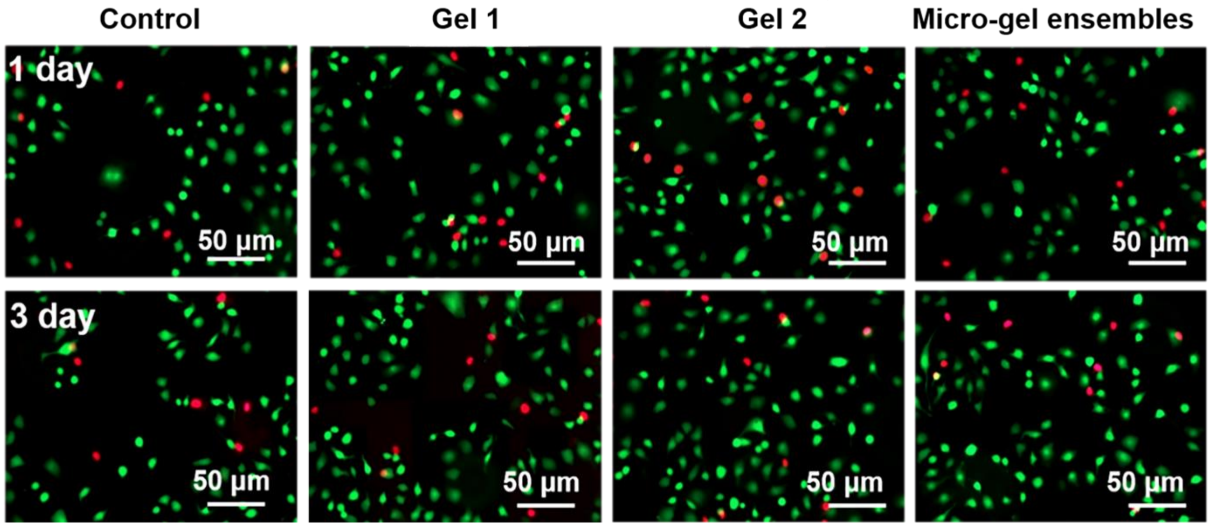

Figure S18. Live/Dead staining of L929 fibroblasts on control sample (blank), Gel **1**, Gel **2** and micro-gel ensembles. The scale bars are 50 μm, n = 24/group.

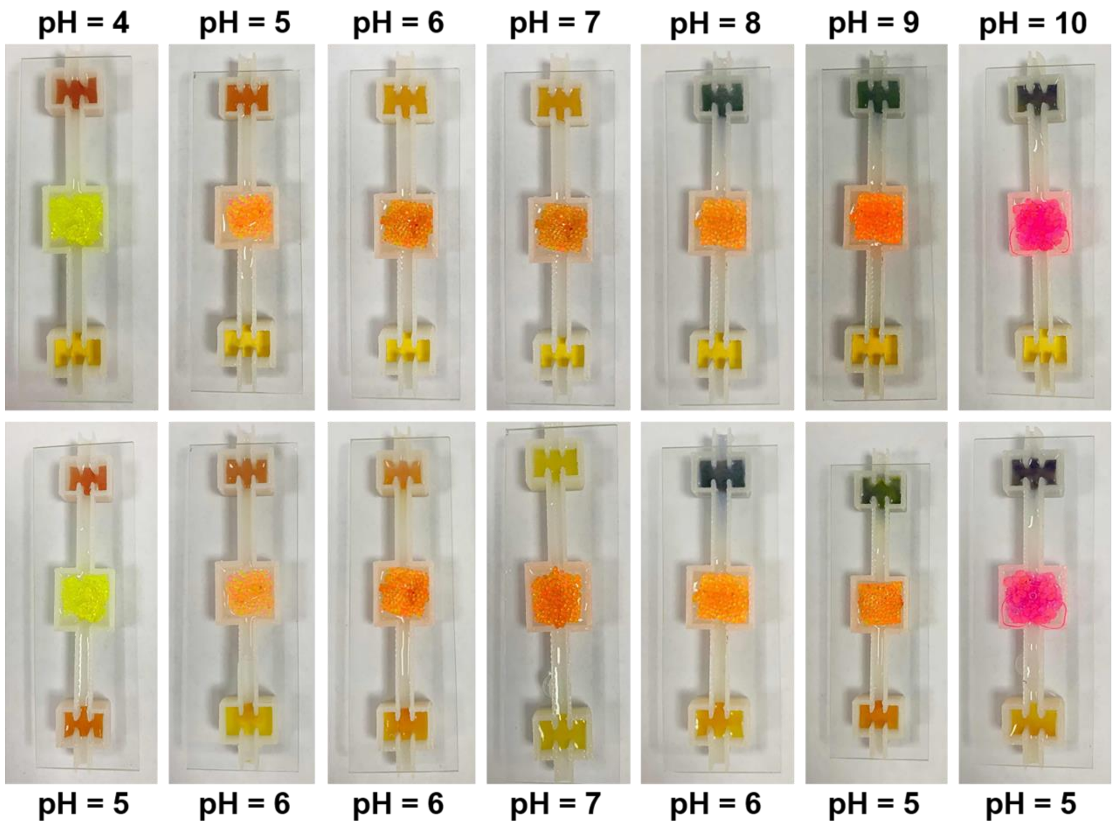

Figure S19. Photographs demonstrating the in vitro chip simulates the microenvironment changes of the wound.

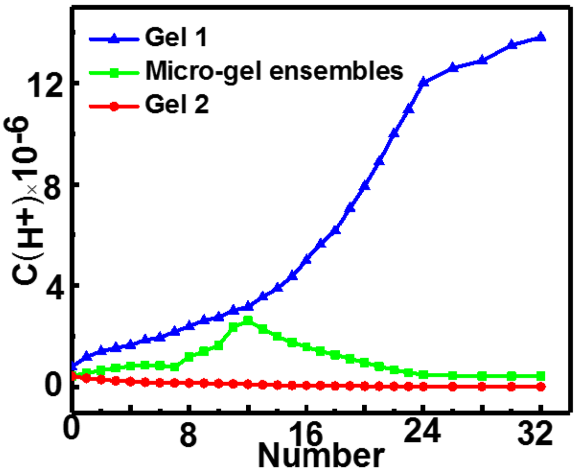

Figure S20. The curve of H^+ concentration with the number of gels (Gel **1**, micro-gel ensembles, Gel **2**).

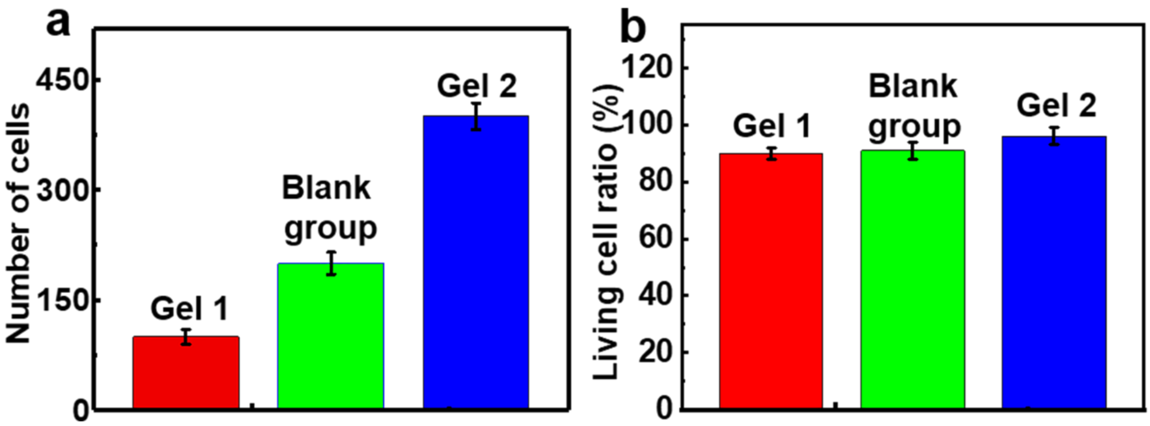

Figure S21. a) The number of fibroblasts and b) living cell ration on wounds treated with different materials. Bars represent standard error, for statistical analysis in a, b, n = 24.

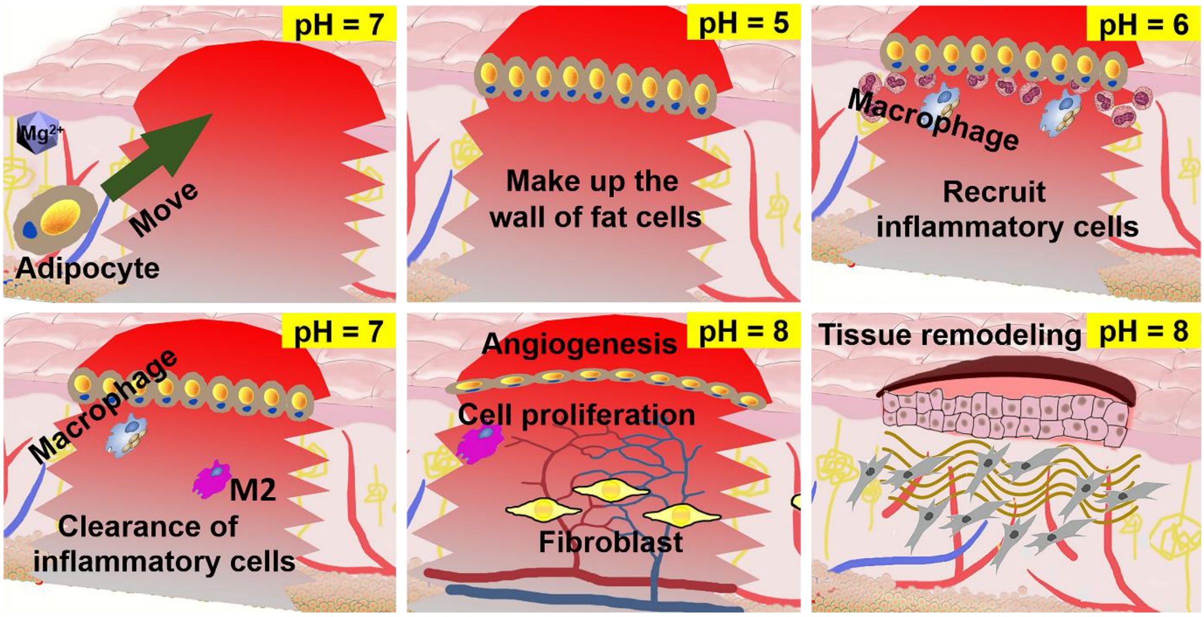

Figure S22. Schematic illustration of wound healing process.

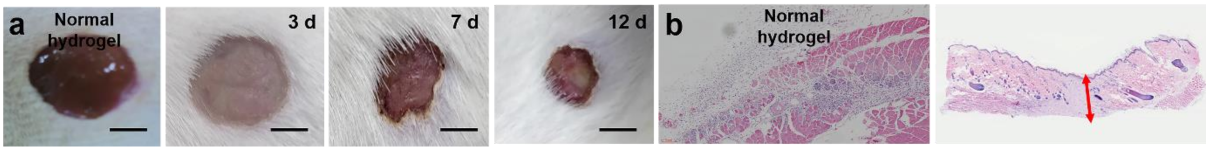

Figure S23. a) Digital photographs of representative skin wound healing processes in rats treated with normal hydrogel. b) Microscopy images of granulation growth for normal hydrogel group by H&E. The scale bars are 1 cm, n = 7/group.

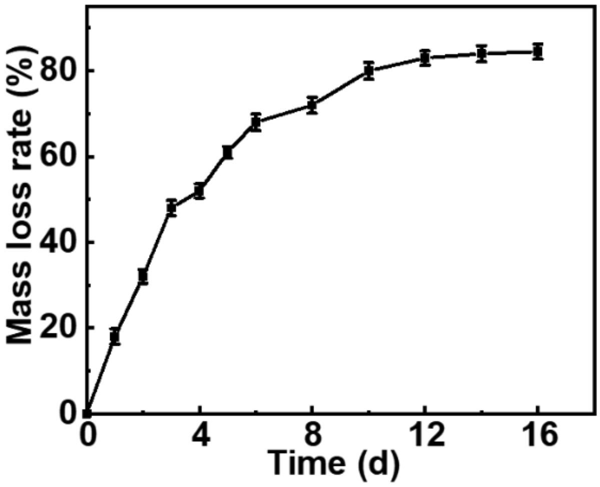

Figure S24. In vitro degradation curve of Gel **2**. Gel **2** gradually losed mass with time. The mass loss on the first day was about 20% of the total mass. After 16 days of degradation, the mass loss of Gel **2** reached 87%. Bars represent standard error, for statistical analysis, n = 3.

Table S1. The relationship between the internal and external flow rate and the size of hydrogel microsbeads.

**V_i= 2mL/h**		**V_o = 30mL/h**		**V_i :V_o =1:20**	
**V_o (mL/h)**	**D (μm)**	**V_I (mL/h)**	**D (μm)**	**V_i :V_o =1:20**	**D (μm)**
**20**	**1602**	**1**	**1301**	**1:20**	**1200**
**40**	**1250**	**2**	**1410**	**2:40**	**1160**
**60**	**1109**	**3**	**1545**	**3:60**	**1092**
**80**	**1029**	**4**	**1586**	**4:80**	**1071**
**100**	**917**	**5**	**1667**	**5:100**	**1021**

Table S2. Relationship between different mass ratios (HPA:AA) and healing efficiency of Gel **1** before and after healing.

**Ration (HPA:AA wt/wt)**	**Before the healing (MPa)**	**After healing (MPa)**	**Healing efficiency (%)**
**4:6**	**0.223**	**0.16**	**71**
**5:5**	**0.21**	**0.18**	**85**
**6:4**	**0.25**	**0.23**	**92**
**7:3**	**0.165**	**0.12**	**78**
**3:7**	**0.145**	**0.11**	**75**

Table S3. Swelling performance of Gel **1** in different time periods.

**Time (h)**	**0**	**1**	**2**	**3**	**4**	**5**	**6**	**7**
**Swelling ratio (%)**	**0**	**102**	**159**	**206**	**253**	**284**	**315**	**364**

Table S4. Swelling performance of Gel **2** in different time periods.

**Time (h)**	**0**	**1**	**2**	**3**	**4**	**5**	**6**	**7**
**Swelling ratio (%)**	**0**	**89**	**145**	**186**	**212**	**243**	**278**	**302**

Table S5. The relationship of H^+ concentration with the number of gels (Gel **1,** Gel **2,** Micro-gel ensembles).

	**Number**	**4**	**8**	**12**	**16**	**20**	**24**	**28**	**32**
**Gel 1**	**pH**	**6.5**	**6.1**	**5.8**	**5.6**	**5.3**	**5.1**	**5**	**4.9**
	C_H^+	$10^{-6.5}$	$10^{-6.1}$	$10^{-5.8}$	$10^{-5.6}$	$10^{-5.3}$	$10^{-5.1}$	10^{-5}	$10^{-4.9}$
**Gel 2**	**pH**	**7.5**	**7.8**	**8**	**8.3**	**8.9**	**9.1**	**9.5**	**9.7**
	C_H^+	$10^{-7.5}$	$10^{-7.8}$	10^{-8}	$10^{-8.3}$	$10^{-8.9}$	$10^{-9.1}$	$10^{-9.5}$	$10^{-9.7}$
**Micro-gel ensembles**	**pH**	**6.8**	**6.5**	**6.1**	**6.8**	**7.4**	**7.5**	**7.2**	**7.2**
	C_H^+	$10^{-6.8}$	$10^{-6.5}$	$10^{-6.1}$	$10^{-6.8}$	$10^{-7.4}$	$10^{-7.5}$	$10^{-7.2}$	$10^{-7.2}$
